# The immunoregulation of mesenchymal stem cells plays a critical role in improving the prognosis of liver transplantation

**DOI:** 10.1186/s12967-019-02167-0

**Published:** 2019-12-10

**Authors:** Chenxia Hu, Lanjuan Li

**Affiliations:** 1 Collaborative Innovation Center for Diagnosis and Treatment of Infectious Diseases, State Key Laboratory for Diagnosis and Treatment of Infectious Diseases, First Affiliated Hospital, School of Medicine, Zhejiang University, Hangzhou, Zhejiang People’s Republic of China; 2 National Clinical Research Center for Infectious Diseases, The First Affiliated Hospital, School of Medicine, Zhejiang University, Hangzhou, Zhejiang People’s Republic of China

**Keywords:** Mesenchymal stromal cell, Immunoregulation, Liver transplantation, Rejection, Prognosis

## Abstract

The liver is supplied by a dual blood supply, including the portal venous system and the hepatic arterial system; thus, the liver organ is exposed to multiple gut microbial products, metabolic products, and toxins; is sensitive to extraneous pathogens; and can develop liver failure, liver cirrhosis and hepatocellular carcinoma (HCC) after short-term or long-term injury. Although liver transplantation (LT) serves as the only effective treatment for patients with end-stage liver diseases, it is not very popular because of the complications and low survival rates. Although the liver is generally termed an immune and tolerogenic organ with adaptive systems consisting of humoral immunity and cell-mediated immunity, a high rejection rate is still the main complication in patients with LT. Growing evidence has shown that mesenchymal stromal cell (MSC) transplantation could serve as an effective immunomodulatory strategy to induce tolerance in various immune-related disorders. MSCs are reported to inhibit the immune response from innate immune cells, including macrophages, dendritic cells (DCs), natural killer cells (NK cells), and natural killer T (NKT) cells, and that from adaptive immune cells, including T cells, B cells and other liver-specific immune cells, for the generation of a tolerogenic microenvironment. In this review, we summarized the relationship between LT and immunoregulation, and we focused on how to improve the effects of MSC transplantation to improve the prognosis of LT. Only after exhaustive clarification of the potential immunoregulatory mechanisms of MSCs in vitro and in vivo can we implement MSC protocols in routine clinical practice to improve LT outcome.

## Background

The liver is supplied by a dual blood supply, including the portal venous system and the hepatic arterial system; thus, the liver organ is exposed to multiple gut microbial products, metabolic products, and toxins; is sensitive to extraneous pathogens; and can develop liver failure, liver cirrhosis and hepatocellular carcinoma (HCC) after short-term or long-term injury. Early in 1963, the first case of liver transplantation (LT) was performed by Dr. Thomas Starzl for irreversible injury, but it was not very popular because of the complications and low survival rates throughout the 1960s and 1970s [1]. Although the liver is generally termed an immune and tolerogenic organ with adaptive systems consisting of humoral immunity and cell-mediated immunity, a high rejection rate is still the main complication in patients with LT [2]. Moreover, acute graft-versus-host disease, which is induced by the interaction of the innate and adaptive immune systems, is a serious and life-threatening complication of LT that occurs in 1% to 2% of liver allograft recipients. Thus, therapies targeting immune cells may be beneficial for transplanted grafts and protect against severe rejection processes. Although other factors, such as secondary infection and unstable surgical techniques, also influence liver graft and patient survival, the main issue is the determination of safe and effective immunosuppression agents. Cyclosporine emerged as an effective immunosuppressant that obviously reduced the rejection rate and prolonged the survival time of LT recipients [3]. However, the application of immunosuppressive agents contributes to metabolic complications, inevitable viral recurrence, and opportunistic infections in LT recipients [4].

Growing evidence has shown that mesenchymal stromal cell (MSC) transplantation could serve as an effective immunomodulatory strategy to induce tolerance in various immune-related disorders. The ISCT committee set a definition of MSCs as follows: MSCs are plastic-adherent and fibroblast-like after culture in vitro; they are positive for surface molecules such as CD105, CD73 and CD90 but negative for surface molecules such as CD45, CD34, CD14 (or CD11b), CD79alpha (or CD19) or human leukocyte antigen (HLA)-DR by flow cytometry; and they can be differentiated into adipocytes, osteocytes and chondrocytes in vitro [5]. These multipotent cells are generally isolated from various tissues, including bone marrow, adipose, umbilical cord, tooth pulp, and cord and participate in the regulation of organ homeostasis, tissue remodeling and damage repair [6]. They are immune-privileged in vivo since they have low expression of class II major histocompatibility complex (MHC)-II and costimulatory molecules [7]. MSCs are able to migrate into injured liver sites, undergo proliferation and hepatic differentiation, secrete anti-inflammatory factors and interact with immune cells to repair liver injury and prohibit liver failure [8]. Intriguingly, MSCs participate in generating a balanced microenvironment via cell–cell interactions and paracrine pathways. Thus, MSC transplantation serves as a novel treatment regimen for preventing graft rejection and treating autoimmune diseases such as graft-versus-host disease via their immunomodulatory effects [9].

In this review, we summarized the relationship between LT and immunoregulation, and we focused on how to improve the effects of MSC transplantation to improve the prognosis of LT. Then, we highlight that the time points of MSC administration or preconditioning with anti-inflammatory factors or gene modification further improve the effects of MSC-based therapies in LT grafts or recipients. Only after exhaustive clarification of the potential mechanisms of MSCs on immunoregulation in vitro and in vivo can we implement MSC protocols in routine clinical practice to improve LT outcome.

## Activation of innate or adaptive immune cells in vivo

Both innate immune cells and adaptive immune cells generated from bone marrow-derived progenitor cells constitute the immune system in mammals. In response to external pathogens, innate immune cells respond more rapidly than adaptive immune cells. Pattern recognition receptors and cytokine receptors quickly activate innate immune cells to lyse foreign pathogens and generate various cytokines for further activation of adaptive immune cells.

### Activation of innate immune cells in vivo

The innate immune system consists of macrophages, dendritic cells (DCs), natural killer cells (NK cells), and natural killer T (NKT) cells. All these immune cells are able to activate the adaptive immune cells through cell–cell interaction and secretion of chemokines and cytokines [10]. Macrophages are immune cells that can be generated from tissue resident macrophages and circulating macrophages from bone marrow [11]. Macrophages can be polarized into proinflammatory M1 or anti-inflammatory M2 macrophages. M1 macrophages produce multiple inflammatory factors, including tumor necrosis factor (TNF)-α, interleukin (IL)-1β, CCL2, and CXCL1, to generate and promote tissue damage after they are activated by toll-like receptor (TLR) agonists, pathogen-associated molecular patterns (PAMPs) and damage-associated molecular patterns (DAMPs) in response to changed microenvironments [12]. M1 macrophages will switch to IL-10-secreting M2 macrophages to decrease tissue injury and resolve inflammation in response to dead cells and several inflammatory factors, including IL-4 and IL-13 [13]. DCs are important innate immune cells that serve as antigen-presenting cells (APCs) to present and process external antigens to secondary lymphoid tissues, such as the spleen and lymph nodes, for further activation of adaptive immune cells. The DCs are divided into conventional DCs and plasmacytoid DCs under steady state in vivo, while they change their phenotype and functionality according to different inflammatory conditions. Although the immune function of plasmacytoid DCs is weaker than that of mature DCs, plasmacytoid DCs are a subset of DCs that can produce IL-10 and TGF-β to induce the differentiation of T cells into Tregs via the TLR7/9 pathway [14]. Activated DCs selectively secrete various cytokines, including IL-12, IL-23 and Notch ligands, in response to the inflammatory microenvironment to induce the differentiation of activated T cells into T helper 1 (Th1), Th2 and Th17 cells [15]. In response to inflammatory stimulation, the quiescent and tolerant DCs will transform into inflammatory DCs and induce T cell-mediated immune responses [16]. Intake of apoptotic DCs enabled the conversion of immature DCs into tolerogenic DCs to promote the differentiation of T cells into forkhead box P3 (Foxp3)+ Tregs [17]. NK cells are innate immune cells that directly kill injured cells by natural cytotoxicity and secretion of interferon-gamma (IFN-γ). NK cells are able to distinguish self- and non-self for innate and adaptive immune responses and participate in the lysis of harmful pathogens in allografts [18]. Intriguingly, NKT cells are a type of noncirculating, tissue-resident lymphocyte with innate immunity and adaptive immunity. These specific immune cells express both the T cell receptor (TCR) α-chain and the surface receptors of NK cells for the regulation of immunoregulation [19]. NKT cells recognize lipids present on CD1d and secrete various cytokines after differentiation into distinct subsets that resemble subsets of CD4+ T helper cells including Th1, Th2 and Th17 cell subsets and subsets of innate lymphocyte cells (ILCs). Activated NKT1 cells are similar to Th1 cells and group 1 ILCs (ILC1s), which express high levels of T-bet, IL-4 and IFN-γ. NKT2 cells are similar to Th2 cells, which secrete high levels of cytokines, including IL-4, IL-5 and IL-13, and NKT17 cells resemble Th17 cells, which secrete high levels of cytokines including IL-17, IL-22, granulocyte macrophage colony stimulating factor (GM-CSF) and TNF [20].

### Activation of adaptive immune cells in vivo

In comparison to the innate immune system, the activation of T and B cells leads to selective expression of several types of antigen receptors that shape their specific functions in immunoregulatory processes. Moreover, TCR specifically binds antigens in the context of MHC molecules, while B cell antigen receptor (BCR) binds antigens in an MHC-independent manner. The costimulatory receptor engagement maximally activates the innate and adaptive immune systems to clear pathogens and respond rapidly to reinfection [21]. In addition, TCRs and other costimulatory factors are responsible for the activation of naïve T cells in vivo, thus protecting against infection-induced cytotoxicity and immune-mediated diseases. Different stimulations will promote the differentiation of activated CD4+ T cells into T helper cells and regulatory T cells (Tregs) [22]. On the other hand, multiple extraneous pathogens transform CD8+ T cells into cytotoxic T lymphocytes, which promote the lysis of injured cells via secretion of granzymes, perforins, and cytokines [23]. Albano et al. [24] demonstrated that CD4+ T helper cells were indispensable for activation of cytotoxic CD8+ T cells and B lymphocytes and differentiation of macrophages to eliminate cells infected with external pathogens. B cells, a subset of lymphocytes from bone marrow, take part in the regulation of T-cell-dependent and T-cell-independent immunoregulation. B cells effectively recognized exotic antigens and then proliferated and differentiated into antibody-producing B lymphocytes and memory B lymphocytes to protect against pathogen-induced injury in vivo [25]. B lymphocytes are divided into B1 cells, which are enriched in the pleural and peritoneal cavities, and B2 cells, which are conventional B lymphocytes. Moreover, regulatory B cells (Bregs) are another subset of B lymphocytes that can produce IL-10 and participate in the induction of tolerance in vitro and in vivo [26].

## Regulation of immune cells in response to LT

The liver is an immunotolerant tissue with various cells expressing low levels of MHC antigens, and it is difficult to induce an innate or adaptive immune response in the liver [27, 28]. Multiple immune cells, such as T cells, B cells, NK cells, NKT cells, liver sinusoidal endothelial cells (LSECs), Kupffer cells (KCs) and DCs, are located in liver tissue and migrate from peripheral blood for recognition and response to pathogens in an antigen-specific manner. Intrahepatic immune cells exert high immunosuppressive effects via cell–cell interactions and secretion of immunosuppressive cytokines after LT.

Two sources of immune cells, donor liver-resident cells and recipient immune cells, respond to the altered microenvironment after LT. The former immune cells are those graft-derived immune cells that enter the peripheral blood of recipients, and the latter are those recipient-derived immune cells that invade into liver grafts [29]. T cells and B cells first recognize pathogens and then take part in antigen-presenting activities. Allospecific T cells recognize foreign MHC molecules on donor tissue cells and play a critical role in the rejection of solid organ grafts [30]. Kim et al. [31] showed that calcineurin inhibitor-based immunosuppression maintained effector T or memory B cells during the early posttransplantation period accompanied by suppression of Tregs.

In normal adult liver tissue, NK cells are the most abundant immune cells in all liver-derived lymphocytes. NK cells may participate in the induction of immune tolerance, as García et al. [32] showed that recipients with immune tolerance have higher levels of NK cells than those with immune rejection in their peripheral blood. The liver graft-derived recipient NK cells generate tolerant phenotypes after decreasing receptors, cytotoxicity and cytokine secretion via perturbation of the IL-12/STAT-4 signaling pathway after LT [33]. However, there is debate about the effects of NK cells on LT recipients. Depletion of NK cells or inhibition of IFN significantly upregulated the survival rate of liver grafts; thus, therapy targeting NK cells and their secretion of cytokines may contribute to the improvement in LT prognosis [34]. LSECs that constitute the wall of the hepatic sinusoid are the main nonparenchymal cells in liver tissue, while there are only a few reticular fibers attached to them that express only a few of the MHC-II molecules. LSECs switch into proinflammatory and prothrombotic states and vasoconstrict after the excised liver grafts were preserved by cold storage prior to LT. The unabridged functions are critical to the outcome of LT according to the current study [35]. Intriguingly, these nonparenchymal cells interact with other immune cells for antigen presentation and elimination of harmful immune responses in vivo. The antigen presentation of LSECs induced immunological tolerance rather than enhancement of immunoregulation against cytotoxic antigens through CD8+ T cells, thus leading to suppression of the immunological response in liver [36]. LESCs significantly inhibited the proliferation of CD4+ T cells and IL-2 generation, and they further induced the apoptosis of CD4+ T cells via the Fas-FasL pathway [37]. In addition, LSECs further promoted the differentiation of CD4+ T cells into CD25lowFoxp3− Tregs, which decreased the number of infiltrated inflammatory cells in liver tissue [38]. Twenty percent of the nonparenchymal cells in the liver tissue are residential macrophages (KCs), and these cells are located in the liver sinusoid and participate in the phagocytosis process via secretion of cytokines including IL-1, IL-6, IL-12, IL-18, TNF-α, and IFN-γ and presentation of antigens [39, 40]. Liver KCs were reported to activate the TLR2/4 pathway and secrete IL-10 for suppression of IL-18-dependent NK cell activation [41]. Liver graft rejection was related to high levels of IL-2, IFN-γ and TNF-α and low levels of IL-10, while immune tolerance was induced by KCs with high PD-L1 levels; these KCs can directly contact T cells and decrease the proliferation and functions of T cells after LT to reduce acute rejection [42]. KCs are able to promote the apoptosis of T cells through the Fas/FasL pathway [39], and Chen et al. [43] showed that pretreatment with KCs in the recipients before LT significantly decreased the number of apoptotic hepatocytes and attenuated the liver injury content in recipients with LT. In addition, KCs also promote Treg proliferation and secretion of IL-10 directly to inhibit the immune response of cytotoxic T lymphocytes on antigens targeting liver tissue [44]. Liver-derived DCs significantly inhibited the T cell response and promoted Treg activation to generate a definite immune tolerance via secretion of IL-10 [45]. The infusion of Tregs and immature DCs decreased the levels of total bilirubin and alanine aminotransferase and prolonged liver allograft survival of heterotopic LT rats via inducing alloantigen tolerance, as shown by upregulation of IL-10 and transforming growth factor (TGF)-β1 and downregulation of IL-12 [46].

The balance between inflammation and anti-inflammation in liver tissue is responsible for the short- and long-term outcomes of LT. Modification of immune cells by immunosuppressive drugs, cytokines, stem cells or other pathways in vivo may decrease the rejection rate of liver grafts in recipients with LT. Thus, moderate immunosuppression by MSC transplantation with minimal adverse effects may prevent rejection and graft loss in LT recipients.

## The interaction between MSCs and immune cells

**Fig. 1:**
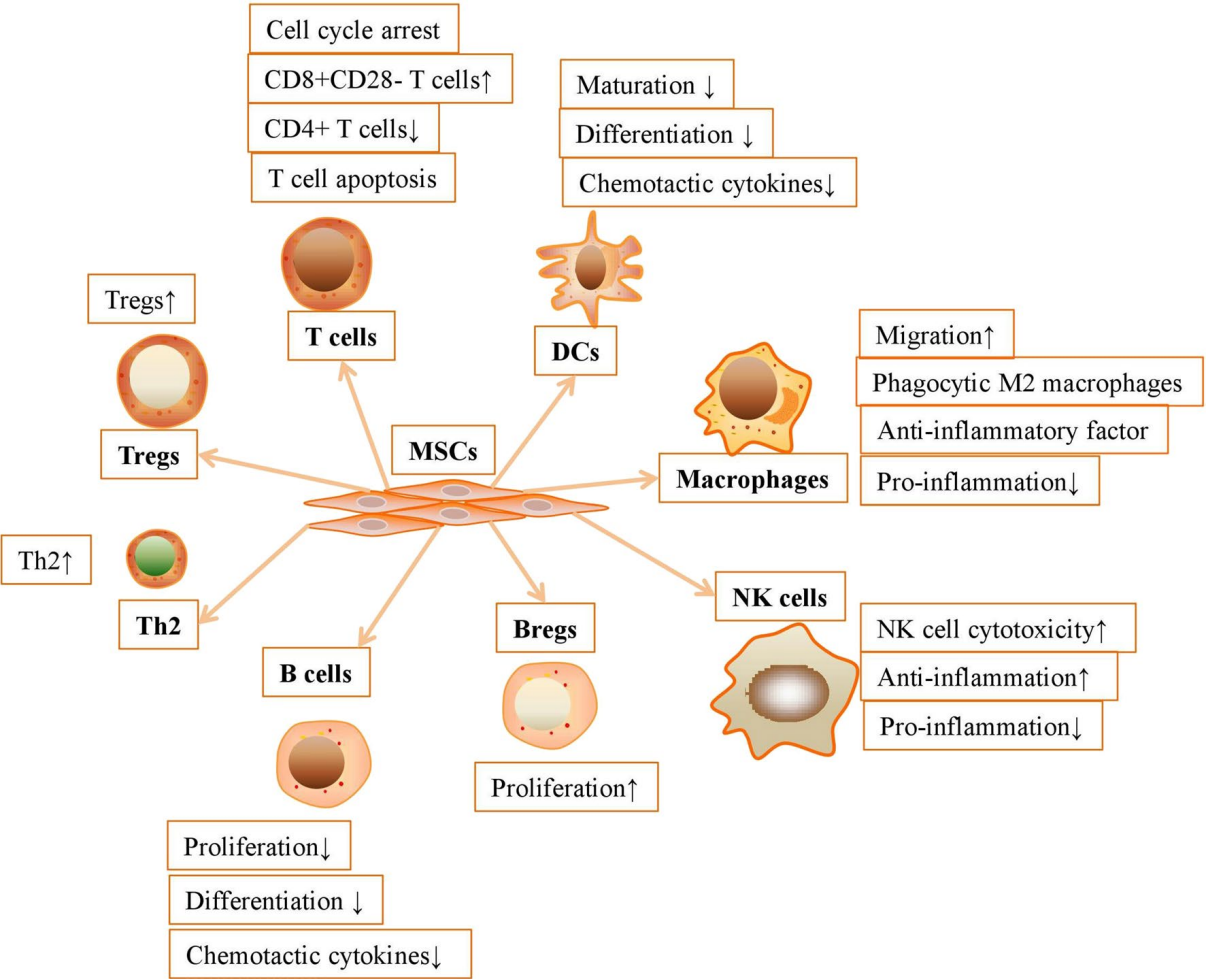
MSCs inhibit the immune response from innate and adaptive immune cells to generate a tolerogenic microenvironment

Mesenchymal stromal cells can establish a stable and balanced microenvironment via regulation of innate or adaptive immune cells. The cell–cell interaction and specific secretome between MSCs and immune cells can guarantee the successful treatment of immune-related diseases. MSCs interact with innate and adaptive immune cells to regulate inflammation in vivo and in vitro. MSCs are reported to inhibit the immune response from macrophages, DCs, NK cells, NKT cells, T cells, Tregs, B cells and Bregs to generate a tolerogenic microenvironment (Fig. 1).

### The interaction between MSCs and innate immune cells

Mesenchymal stromal cells effectively decrease IL-2-induced proliferation, cytotoxicity, and cytokine secretion (IFN-γ, IL-10 and TNF-α) in activated NK cells in an IDO- and PGE2-dependent manner [47–49]. However, Casado et al. [50] highlighted that MSCs can only impair NK cell cytotoxicity via cell–cell contact. On the other hand, MSCs significantly prohibited the expansion, proliferation and IFN-γ secretion in invariant NKT cells [51]. Liu et al. [52] demonstrated that MSCs significantly upregulated the recruitment of macrophages into injured sites for treating immune disorders and enhancing the repair of tissue injury. MSCs with high IDO activity effectively promoted the generation of anti-inflammatory M2 macrophages to block T cell activation; thus, T cell inhibition further amplified the immunosuppressive effects of MSCs [53]. It has been reported that MSCs also inhibited the generation and antigen presentation of peripheral blood monocyte-derived DCs after inhibiting cytokine release, differentiation and maturation of DCs [54, 55]. MSCs and MSC-derived supernatants inhibited the activation and maturation of DCs by downregulating endocytosis and IL-12 production in DCs and then inhibited the activation of alloreactive T cells in vitro [54]. Furthermore, MSCs are able to inhibit the immunogenicity and T cell activation capacity of regulatory DCs, and these DCs can subsequently activate Tregs and secrete the anti-inflammatory factor IL-10 [56].

### The interaction between MSCs and adaptive immune cells

In addition to the innate immune system, MSCs significantly induced cell cycle arrest in T cells after downregulation of cyclin D2 and upregulation of p27kip1 in vitro [57]. MSCs upregulated the number of CD8+CD28− T cells, subsequently inhibiting the proliferation and activation of CD4+ T cells via downregulation of IFN-γ and enhancing the apoptosis of activated CD4+ T cells [58]. The apoptosis of T cells is closely related to the conversion of tryptophan into kynurenine via an IDO-dependent pathway in the presence of IFN-γ [59]. However, several investigators have highlighted that MSCs are only able to suppress T cell activity after pretreatment with inflammatory factors, such as IFN-γ, TNF-α, IL-1α or IL-1β [60]. These preconditioned MSCs participated in immunoregulation by significantly upregulating the expression of inducible nitric oxide synthase (iNOS) and cyclooxygenase 2 for the generation of nitric oxide (NO) and prostaglandin E2 (PGE2) [61]. In addition, MSCs secreted multiple cytokines and chemokines for recruitment of T cells into injured sites for immunosuppression and injury repair [62]. Furthermore, MSCs are reported to exert their immunoregulatory capacity by Treg induction indirectly, as Prevosto et al. [63] showed that MSCs significantly inhibited T lymphocytes after promoting the generation of Tregs from CD4+ or CD8+ T lymphocytes. However, Jiang et al. [64] debated that depletion of CD4+ CD25+ Tregs did not affect the immunosuppression of MSCs on CD4+ T cells, and they concluded that MSCs were not as important in Treg regulation. In a coculture system, MSCs transformed Th1 cells with high levels of proinflammatory factors (IL-2 and IFN-γ) into Th2 cells with high levels of anti-inflammatory factors (IL-4 and IL-10) [65].

Mesenchymal stromal cells were reported to inhibit the B lymphocyte-related humoral immune response via blocking the proliferation, differentiation and chemotactic cytokine production of B cells [66]. Peng et al. [67] demonstrated that infusion of MSCs significantly increased the survival time of patients with refractory chronic graft-versus-host disease through IDO-induced IL-10 secretion and Breg proliferation. Moreover, MSCs induced the generation of phagocytic M2 macrophages, which are able to secrete a large amount of anti-inflammatory factor IL-10, accompanied by low levels of inflammatory factors (IL-6, TNF-α and IL-1β) [68, 69].

## MSC transplantation for improvement in LT prognosis

Acute rejection is commonly encountered in LT recipients and may impact their long-term survival if rejection is severe or recurrent. Acute rejection after LT is usually treated with large doses of immunosuppressants with severe and toxic side effects, so it is imperative to find a safe and effective method for preventing rejection in LT recipients. Although MSCs significantly suppress the immune response by cell–cell interactions and the secretion of various cytokines after allogenic LT, pretreatments with growth factors or gene modification on MSCs can more obviously improve the prognosis of LT via regulation of the immune system (Table 1).

**Table 1:** The potential immunoregulatory mechanisms of MSCs in improving the prognosis of LT

Species of MSCs	Pretreatment	Source	Dose (number)	Model	Mechanism	Effect	Refs.
Mouse	N/A	Bone marrow	1 × 10^6^	Mouse	Suppress Kupffer cell apoptosis, Th1/Th17 immune responses, chemokine expression and inflammatory cell infiltration	Alleviate liver graft injury; upregulate the survival rate of animals with LT	[70]
Rat	N/A	Bone marrow	2.5 × 10^5^	Rat	Inhibit the proliferation of CD4+ T cells and activation of CD8+ T cells; upregulate the levels of TGF-β1, FoxP3, IL-10, and CTLA-4	Attenuate the rejection rate of LT	[71]
Human	N/A	Umbilical cord	1 × 10^6^/kg body weight	Human	Activate Tregs; inhibit Th17 cells; increase the expression levels of TGF-β1 and PGE2	Decrease the alanine aminotransferase level; improve allograft histology	[72]
Rat	N/A	Bone marrow	1 × 10^7^	Rat	Downregulate the levels of Th1/Th2 ratio-associated cytokines; upregulate IL-10; decrease the expression levels of IL-6, IL-17, IL-23, and TNF-α; increase the expression level of TGF-β; activate Th2 and Treg cells; inhibit the activation of Th1 and Th17 cells	Reduce the acute rejection and improve the survival rate of allogeneic LT recipients	[73]
Rat	N/A	Bone marrow	2 × 10^6^	Rat	Activate CD4+CD25+Foxp3+ Tregs	Inhibit allograft rejection; prolong the survival time of LT rats	[74]
Rat	N/A	Bone marrow	1 × 10^6^/200 g	Rat	Reduce liver graft rejection and IL-12 levels; upregulate the levels of TGF-α_1_ and IL-10	Improve liver functions and survival times of rats with LT	[75]
Rat	N/A	Adipose	2.0 × 10^6^	Rat	Downregulate liver impairment and hepatocyte apoptosis; upregulate peripheral Tregs; elevate the expression levels of PCNA, IL-10 and TGF-β1; decrease the expression levels of IL-2 and IL-17	Reduce rejection rate; prolong survival time of the allograft	[76]
Rat	N/A	Bone marrow	2 × 10^6^	Rat	Upregulate PD-L1 expression; downregulate miR-17-5p	Eliminate liver allograft rejection; improve the median survival time of LT recipients	[77]
Swine	N/A	Adipose	1.0 × 10^6^	Rat	Hepatogenic differentiation of MSCs	Protect the function of liver grafts from warm ischemia/reperfusion injury; improve the viability of liver grafts	[82]
Rat	IFN-γ	Bone marrow	5 × 10^6^	Rat	Upregulate the levels of PDL-1, MHC-I, MHC-II, and CD54 and boost the immunosuppressive ability of MSCs	Alleviate acute immunologic rejection of liver grafts	[83]
Rat	Overexpression of TGF	Bone marrow	5 × 10^6^	Rat	Activate Tregs; inactivate Th17 cells	Prevent rejection of liver grafts; reduce the mortality rate of rats with LT	[84]
Rat	Overexpression of IL-10	Bone marrow	2.5 × 10^5^	Rat	Increase the expression of RORγt; decrease the expression level of FoxP3 in MSCs; downregulate the secretion of cytokines such as IL-17, IL-23, IL-6, IFN-γ and TNF-α; upregulate the secretion of cytokines such as IL-10 and TGF-β1	Prolong the mean survival time of LT rats; decrease the Banff scheme grading scores of LT rats	[85]
Rat	Overexpression of HO-1	Bone marrow	1 × 10^7^	Rat	Upregulate the levels of anti-inflammatory factors and peripheral Tregs; downregulate the levels of proinflammatory factors and NK cell viability	Upregulate the median survival time; decrease the rejection activity index	[86]
Rat	Overexpression of HO-1	Bone marrow	5 × 10^6^	Rat	Upregulate the levels of autophagy-related proteins; activate the ERK/mTOR signaling pathway	Protect against liver grafts in the reduced-size liver transplantation rat model	[87]
Rat	Overexpression of HO-1	Bone marrow	1 × 10^6^/kg	Rat	Improve the microcirculation of hepatic sinusoids; recover the energy metabolism of damaged hepatocytes	Attenuate the pathological changes and rejection rate of the transplanted liver grafts in LT models	[88]
Rat	Overexpression of HO-1	Bone marrow	5 × 10^6^	Rat	Upregulate the levels of IL-10 and TGF-β; downregulate the levels of IL-2, IL-6, IL-17, IL-23, TNF-α, and IFN-γ	Improve the liver functions and survival rates of LT recipients; reduce the degree of rejection and apoptotic cells	[89]

### The potential mechanisms of MSCs in improving the survival rate of LT

**Fig. 2:**
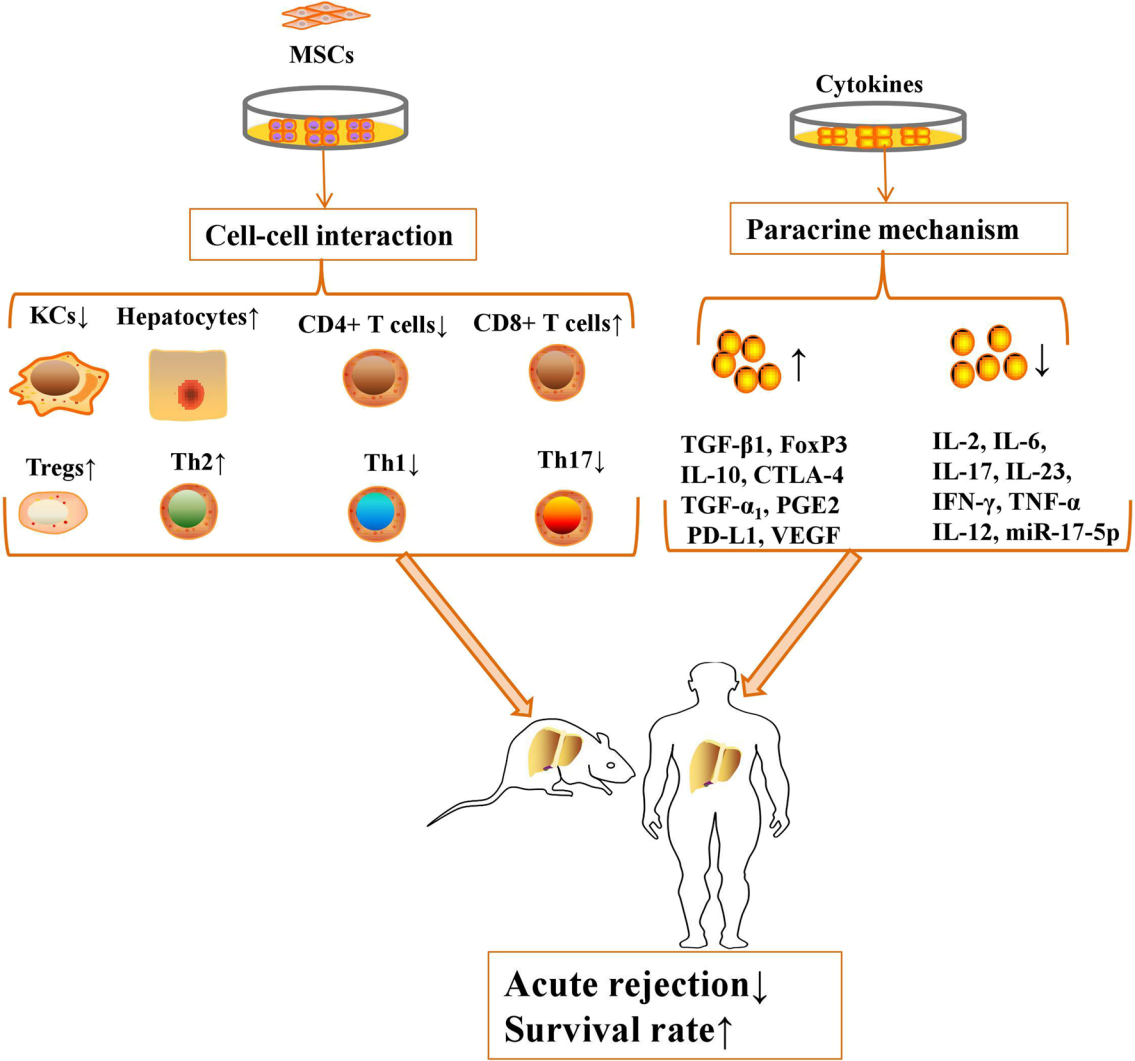
MSCs exert their immunoregulatory capacities to suppress the immune response by cell–cell interactions and secretion of various cytokines after allogenic LT

Currently, MSCs exert their immunoregulatory capacities to suppress the immune response by cell–cell interactions and the secretion of various cytokines after allogenic LT (Fig. 2).

Mesenchymal stromal cells significantly upregulated the survival rate of animals with LT via suppression of KC apoptosis, hepatocyte apoptosis, Th1/Th17 immune responses, chemokine expression and inflammatory cell infiltration [70]. MSCs are reported to inhibit the proliferation of CD4+ T cells and activation of CD8+ T cells in LT recipients on postoperative day 7 [71]. MSC transplantation markedly decreased the alanine aminotransferase level and improved allograft histology via activation of Tregs and Th2 cells and inhibition of Th1 and Th17 cells in animals with LT [72, 73]. Moreover, MSC administration inhibited allograft rejection and prolonged the survival time of LT rats via activation and expansion of CD4+CD25+Foxp3+ Tregs [74].

In addition to the immunoregulation of liver immune cells in vivo, MSC transplantation protects recipients of LT from acute rejection-induced injury via paracrine mechanisms. MSC administration upregulated the levels of TGF-β1, Foxp3, IL-10, and CTLA-4 on postoperative day 7 in rats with LT, while it downregulated the levels of TGF-β1 and Foxp3 on postoperative day 100 compared with those on postoperative day 7 [71]. MSCs improved liver function and the survival time of rats with LT via downregulation of IL-12 but upregulation of IL-10, TGF-α_1_, TGF-β1 and PGE2 [72, 75, 76]. Furthermore, MSCs significantly reduced the acute rejection rate and improved the survival rate of allogeneic LT recipients via downregulating the levels of IL-2, IL-6, IL-17, IL-23, IFN-γ and TNF-α [73, 76]. Chen et al. [77] showed that MSCs significantly eliminated liver allograft rejection and improved the median survival time of LT recipients via upregulation of PD-L1 expression and downregulation of miR-17-5p. On the other hand, injection of MSC-derived conditioned medium inhibited the cell death of LSECs and hepatocytes and promoted liver regeneration, consequently providing additional benefits to the survival rate of rats with reduced-size liver transplantation (RSLT) via downregulating KC activation, neutrophil infiltration and secretion of inflammatory factors while upregulating the levels of vascular endothelial growth factor (VEGF) and matrix metallopeptidase 9 in the liver grafts [78]. In addition, MSC-derived extracellular vesicles have been proved to exert comparable therapeutic capacities as MSCs themselves in recent years. MSC-derived extracellular vesicles are able to promote the proliferation of endogenous stem cells and tissue regeneration [79]. Furthermore, MSC-derived extracellular vesicles are able to transport noncoding RNAs for the regulation of matrix remodeling, epithelial mesenchymal transitions, resolution of inflammation and immune alleviation [80]. Although there are no studies about the effects of MSC-derived extracellular vesicles in improving LT prognosis via their anti-inflammatory effects, MSC-derived extracellular vesicles are worth recognizing as an emerging therapy for treating LT-related immune rejection.

### Management of MSC administration in recipients with LT

Although current studies together showed that MSCs effectively improved LT prognosis after the regulation of immune systems in vivo, the management of MSCs may further improve the therapeutic effects on rejection in liver grafts and recipients. Early treatment with MSC transplantation significantly improved the survival time of rats with acute graft-versus-host disease after LT via upregulation of Treg ratios and Foxp3-positive cells, while late treatment with MSC transplantation from day 8 to day 14 did not attenuate the typical symptoms in rats with acute rejection after LT [81]. This indicates that the time point of MSC transplantation may contribute to the prognosis of LT. To overcome the scarce source of liver grafts, liver donation after cardiac death (DCD) is an alternative approach that may expand the donor pool, while these excised grafts face challenges such as graft dysfunction, early graft loss, and cholangiopathy. Moreover, DCD liver grafts are no longer eligible for transplantation after their warm ischemic time exceeds 30 min. Administration of MSCs protected against warm ischemia–reperfusion injury and protected liver functions of liver grafts from DCD [82].

It is worth highlighting that preconditioning with cytokines or gene modification may enhance the immunoregulatory capacity of MSCs to regulate immune cells in vitro and in vivo. Pretreatment or gene modification of MSCs may serve as an effective method to further improve the immunoregulatory capacity of MSCs in LT. For example, IFN-γ pretreated MSCs had upregulated levels of PDL-1, MHC-I, MHC-II, and CD54 and boosted immunosuppressive ability in vivo, consequently improving the homing capacity of MSCs into the liver tissue to alleviate acute immunologic rejection of liver grafts in rats [83]. Transplantation of TGF-overexpressing MSCs also prevented rejection of liver grafts and reduced the mortality rate of rats with LT via activation of Tregs and inactivation of Th17 cells [84]. On the other hand, IL-10-overexpressing MSCs significantly prolonged the mean survival time while decreasing the Banff scheme grading scores of LT rats via upregulation of retinoid acid receptor-related orphan receptor gamma t (RORγt) and downregulation of Foxp3 in MSCs in a time-dependent manner. IL-10 overexpression also downregulated the secretion of cytokines such as IL-6, IL-17, IL-23, IFN-γ and TNF-α while upregulating the secretion of cytokines such as IL-10 and TGF-β1 in MSCs [85]. Moreover, several studies have indicated that heme oxygenase-1 (HO-1) is a key gene in enhancing the immunoregulatory capacity of MSCs in vivo. HO-1-overexpressing MSCs upregulated the median survival time and decreased the rejection activity index via the upregulation of anti-inflammatory factors and peripheral Tregs and downregulation of proinflammatory factors and NK cell viability [86]. HO-1-overexpressing MSCs also protected liver grafts against injury in the RSLT rat model via the upregulation of autophagy-related proteins through the ERK/mTOR signaling pathway [87]. Furthermore, overexpression of HO-1 in MSCs improved the microcirculation of hepatic sinusoids and recovered the energy metabolism of damaged hepatocytes via improving the activities of mitochondrial aspartate aminotransferase and ATPase [88]. The immunoregulation of HO-1-overexpressing MSCs is also mediated by the upregulation of IL-10 and TGF-β and the downregulation of IL-2, IL-6, IL-17, IL-23, TNF-α, and IFN-γ to improve allogeneic LT outcomes [89].

To this end, pretreatments with growth factors or gene modification are insufficient to clarify the potential effects of MSC preconditioning on reducing LT-related rejection, due to the scarcity of studies. Thus, it is still obligatory to expand the related studies and explore the potential mechanisms to improve the therapeutic effects of MSCs in the attenuation of graft-versus-host diseases in liver tissue.

## Conclusion

Several types of APCs in the liver and blood circulation recognize external pathogens and subsequently initiate defensive immune mechanisms to attenuate liver inflammation and liver injury. MSCs have been shown to regulate the immune system to attenuate liver inflammation and participate in promoting internal environment homeostasis for the generation of tolerogenic microenvironments in vivo. Striking evidence suggests that MSCs participate in the immunoregulation of liver grafts and recipients to improve the prognosis of LT in preclinical models via cell–cell interactions and anti-inflammatory cytokines. However, pretreatment with anti-inflammatory factors or gene modification of MSCs can further improve the therapeutic effects of MSCs in alleviating the acute graft-versus-host rate. In addition, most gene modifications are overexpression of key genes to enhance the immunoregulatory capacity of MSCs. It is worth considering that knockdown of specific genes in MSCs may also contribute to the improvement of LT prognosis. However, gene modification of MSCs may induce tumorigenesis in recipients after long-term treatment. In the near future, MSC transplantation will further permits a reduction in drug burden and pharmacotherapy inhibitor-associated side effects in LT recipients. However, verification in large-scale and prospective clinical trials has the potential to lead to implementation of MSC protocols in clinical practice for improving LT outcomes.

## Data Availability

Not applicable.

## Abbreviations

HCC: hepatocellular carcinoma
LT: liver transplantation
MSC: mesenchymal stem cell
HLA: human leukocyte antigen
MHC: major histocompatibility complex
DCs: dendritic cells
NK cells: natural killer cells
NKT: natural killer T
TNF: tumor necrosis factor
IL: interleukin
TLRs: toll-like receptors
PAMPs: pathogen-associated molecular patterns
DAMPs: damage-associated molecular patterns
APCs: antigen-presenting cells
IFN-γ: interferon-gamma
TCR: T cell receptor
Th1: T helper 1
ILCs: innate lymphocyte cells
ILC1s: group 1 ILCs
GM-CSF: granulocyte-macrophage colony-stimulating factor
BCR: B cell antigen receptor
Tregs: regulatory T cells
Bregs: regulatory B cells
LSECs: liver sinusoidal endothelial cells
KCs: Kupffer cells
TGF: transforming growth factor
iNOS: inducible nitric oxide synthase
NO: nitric oxide
PGE2: prostaglandin E2
RSLT: reduced-size liver transplantation
VEGF: vascular endothelial growth factor
DCD: donation after cardiac death
HO-1: heme oxygenase-1
RORγt: retinoid acid receptor-related orphan receptor gamma t
